# Evaluating the Metabolic Basis of α-Gal A mRNA Therapy for Fabry Disease

**DOI:** 10.3390/biology13020106

**Published:** 2024-02-08

**Authors:** Zhendong Zhang, Qi Liu, Zhiwen Deng, Jun Liu, Shuang Li, Mei Hong, Yucai Peng

**Affiliations:** 1 Liverna Therapeutics Inc., Zhuhai 519000, China; zhangzhendong@live-rna.com (Z.Z.); liuqi@live-rna.com (Q.L.);; 2 College of Life Sciences, South China Agricultural University, Guangzhou 510642, China

**Keywords:** Fabry disease, α-galactosidase A, globotriaosylsphingosine, mRNA, metabolomics

## Abstract

**Simple Summary:**

Although mRNA injection-based protein supplementation has emerged as a feasible strategy for treating Fabry disease, whether the administration of lipid nanoparticle-encapsulated mRNA results in the alteration of the metabolome in the in vivo system remains largely unknown. In the present study, an mRNA encoding α-Gal A was developed and exhibited efficient therapeutic effects. Moreover, metabolomics analysis revealed significant alterations in metabolites related to arachidonic acid metabolism, amino acid metabolism, and glycolysis/gluconeogenesis pathways.

**Abstract:**

mRNA injection-based protein supplementation has emerged as a feasible treatment for Fabry disease. However, whether the introduction of LNP-encapsulated mRNA results in the alteration of metabolomics in an in vivo system remains largely unknown. In the present study, α-galactosidase A (α-Gal A) mRNA was generated and injected into the Fabry disease mouse model. The α-Gal A protein was successfully expressed. The level of globotriaosylsphingosine (Lyso-Gb3), a biomarker for Fabry disease, as well as pro-inflammatory cytokines such as nuclear factor kappa-B (NF-κB), interleukin 6 (IL-6), and tumor necrosis factor-α (TNF-α), were greatly decreased compared to the untreated control, indicating the therapeutic outcome of the mRNA drug. Metabolomics analysis found that the level of 20 metabolites was significantly altered in the plasma of mRNA-injected mice. These compounds are primarily enriched in the arachidonic acid metabolism, alanine, aspartate and glutamate metabolism, and glycolysis/gluconeogenesis pathways. Arachidonic acid and 5-hydroxyeicosatetraenoic acid (5-HETE), both of which are important components in the eicosanoid pathway and related to inflammation response, were significantly increased in the injected mice, possibly due to the presence of lipid nanoparticles. Moreover, mRNA can effectively alter the level of metabolites in the amino acid and energy metabolic pathways that are commonly found to be suppressed in Fabry disease. Taken together, the present study demonstrated that in addition to supplementing the deficient α-Gal A protein, the mRNA-based therapeutic agent can also affect levels of metabolites that may help in the recovery of metabolic homeostasis in the full body system.

## 1. Introduction

Fabry disease is the second-most prevalent lysosomal storage disorder (LSD) after Gaucher disease [1]. The disease is caused by mutations in the alpha-galactosidase (*GLA*) gene, which result in a deficiency of the encoded lysosomal enzyme α-galactosidase A (α-Gal A) [2]. Abnormal enzymatic activity leads to the progressive accumulation of globotriaosylsphingosine (Lyso-Gb3) and other glycosphingolipids in the plasma and various tissues [3]. This ultimately triggers a series of cellular and tissue responses that cause damage to multiple organs, such as the heart, liver, spleen, and kidneys [4].

Enzyme replacement therapy (ERT) and pharmacologic chaperones have been developed for Fabry disease [5,6]. ERT with either agalsidase beta (1 mg/kg every other week) or agalsidase alfa (0.2 mg/kg every other week) is available for the treatment of Fabry patients [7]. However, ERT is also subject to significant drawbacks. For example, considerable clinical variability is observed among treated patients, and efficacy is largely dependent on the age at which the therapy is initiated and the development of antibodies against infused enzymes [8]. Pharmacologic chaperone, on the other hand, is only effective for certain patients with specific mutant isoforms of α-Gal A [6]. As Fabry disease is a genetic disease, recent advances in gene therapy provide more options for treating the disease [9]. In murine and non-human primate models of Fabry disease, the delivery of an mRNA encoding α-Gal A has been shown to reduce Lyso-Gb3 and globotriaosylceramide (Gb3) levels in the heart and kidney [10], suggesting the potential of using the mRNA technique for Fabry disease treatment. Metabolic disorders were observed in Fabry patients, accompanied by some complications such as malignant ventricular arrhythmias, sudden cardiac death, end-stage renal failure, and stroke [11]. Fabry’s disease patients also experience oxidative stress, impaired energy metabolism, changes in membrane lipids, disrupted cell transport, and impaired autophagy [2]. Previous studies with patients injected with mRNA vaccines such as those for COVID-19 have shown metabolome alterations [12]. On the other hand, whether the metabolic profile is changed with the treatment of mRNA injection-based protein supplementation is largely unclear.

In the present study, the LNP-mRNA strategy was adapted by preparing an mRNA encoding α-Gal A for the treatment of Fabry disease in mice. To clarify the mechanistic basis for the effects of such LNP-mRNA treatment, the plasma metabolome was analyzed. A total of 20 differential metabolites, which are mainly enriched in the arachidonic acid metabolism, alanine, aspartate and glutamate metabolism, and tricarboxylic acid cycle pathway, were identified. The results revealed that mRNA therapy can sufficiently reduce inflammation, alter amino acid metabolism and energy metabolic pathways, and alleviate oxidative stress in these Fabry model mice.

## 2. Materials and Methods

### 2.1. Chemicals

Dulbecco’s Modified Eagle Medium (DMEM), glutamine, glucose, fetal bovine serum, and antibiotics were purchased from Gibco (Gibco, Grand Island, NY, USA). Anti-α-Gal A (ab168341, 1:5000), anti-β-actin (ab8226, 1:1000), and secondary antibodies (ab6785, 1:10,000) were purchased from Abcam (Abcam, Cambridge, UK). α-Gal A activity assay kit was purchased from Biovision (Milpitas, CA, USA). Globotriaosylsphingosine (Lyso-Gb3) was purchased from Matreya (Pleasant Gap, PA, USA). Lipofectamine 2000 was purchased from Invitrogen (Carlsbad, CA, USA). Liquid chromatography–mass spectrometry (LC–MS)-grade methanol and MS-grade acetonitrile were purchased from Thermo Fisher Scientific (Waltham, MA, USA). OASIS MCX cartridges (30 mg, 60 mm) were purchased from Waters Corp. (Milford, MA, USA). Formic Acid (98.0% for LC–MS) was purchased from Tokyo Chemical Industry (Shanghai, China). The HEK293 cell line was obtained from the American Type Culture Collection (ATCC). ALT, AST, NF-κB, IL-6, and TNF-α ELISA kits were obtained from Enzyme Link Biotechnology Co., Ltd. (Shanghai, China).

### 2.2. Synthesis and Characterization of mRNA and Lipid Nanoparticles

The mouse version of the *GLA* gene was from the mouse database in the NCBI (accession number NM_013463.3). The *GLA* gene was used in Fabry mouse experiments. The mRNA and LNPs used for this study were produced based on the Liverna Therapeutics platform [13]. Briefly, the mRNA molecules were synthesized using an optimized T7 RNA polymerase-mediated transcription reaction with complete replacement of uridine by N1-methyl-pseudouridine in vitro. The reaction included a DNA template containing the open reading frame of the α-Gal A gene flanked by 5′/3′ untranslated region (UTR) sequences and a poly-A tail. In vitro transcribed (IVT) mRNAs were encapsulated in LNPs according to a modified procedure wherein an ethanolic lipid mixture of ionizable cationic lipids, phosphatidylcholine, cholesterol, and polyethylene glycol-lipid was rapidly mixed with an aqueous solution containing the mRNA products. Analytical characterization of the resultant product was conducted, including the determination of particle size, polydispersity, encapsulation, endotoxin levels, and bioburden.

### 2.3. Cell Culture and Transfection

HEK293 cells were cultured in DMEM supplemented with 2 mM glutamine, 4.5 g/L glucose, 10% fetal bovine serum, and antibiotics. The transfection of these cells with cDNA or mRNA (4 μg) (human version *GLA* gene from the NCBI database with accession number AB019551.1). was performed using Lipofectamine 2000 when cells were 50~70% confluent. In the control group, only Lipofectamine 2000 was used for transfection. Before harvesting, transfected cells were cultured in the same growth medium for 24 h.

### 2.4. Western Blotting

Western blotting was used to assess the expression of α-Gal A and β-actin in HEK293 cells. Briefly, an RIPA buffer was used to extract the total protein from these cells, after which protein levels were quantified with a BCA kit. Total proteins were separated using sodium dodecyl sulfate-polyacrylamide gel electrophoresis (SDS-PAGE) gels and transferred onto polyvinylidene fluoride (PVDF) membranes. The membranes were then incubated with appropriate primary and secondary antibodies, after which protein bands were detected with the G: Box Chemi XRQ Imaging System.

### 2.5. Animal Model Experiments

*GLA* gene knockout (KO) mice were used as Fabry disease model animals. These mice were generated in the Shanghai Model Organisms Center (Shanghai, China) using CRISPR/Cas9 technology. Genotyping was performed using DNA from collected tail samples to confirm the loss of the whole precursor-Gal gene encoding sequence (1400 bp). All mice used in the study were housed and bred in the Shanghai Model Organisms Center (Shanghai, China). During all experiments, mice had free access to clean food and water in a facility with a 12-h light/dark cycle. Animal experiments were authorized by the Animal Care and Use Committee of Shanghai Nanfang Model Biotechnology Co., Ltd (Shanghai, China) (2021-0012). Mice were randomly divided into three groups: a control group (PBS), a low-dose mRNA group (1 mg/kg), and a high-dose mRNA group (3 mg/kg). The mRNA encapsulated by LNP was injected into the mouse body via tail vein injection. The plasma, heart, liver, and kidneys of mice were collected at different times for testing α-Gal A activity and Lyso-Gb3 levels.

Male mice (wild-type) were housed in the Shanghai Model Organisms Center (Shanghai, China). The firefly luciferase mRNA (NCBI accession number M15077.1) encapsulated in LNP (1 mg/kg) was injected into the mouse body via tail vein injection. Fluorescence was observed at different locations and time points (0, 2, 6, 12, 24, 48, and 72 h) using a small animal imager (Clinx, IVScope 8000, Shanghai, China).

### 2.6. α-Gal A Activity Assay

A-Gal A activity assay was performed according to published instructions [10]. Briefly, lysates or plasma from mice were mixed with 2-Methylum-belliferyl-a-D-galactopyranoside and 50 mM N-acetylgalactosamine in 100 mM sodium citrate buffer. The reaction mixture was incubated at 37 °C for 2 h. The reaction was stopped by adding 200 μL of stop solution. Fluorescence detection was then performed at the respective excitation and emission wavelengths of 360 nm and 445 nm. α-Gal A activity was expressed as nmol/h/mL plasma or nmol/h/mg total protein.

### 2.7. Immunohistochemistry

Immunohistochemical (IHC) staining was performed using paraffin-embedded sections of murine heart, liver, and kidney tissues. Slides were deparaffinized, hydrated, and antigen-retrieved, and then treated with primary polyclonal anti-mouse α-Gal A (1:500, Abcam, ab168341) overnight at 4 °C. Samples were then incubated with a secondary antibody (1:1000, Abcam, ab6785) for 1 h at room temperature, after which DAB staining was used to visualize the IHC reaction. Sections were imaged with a digital camera (AxioCam, Carl Zeiss, Inc., Thornwood, NY, USA).

### 2.8. Lyso-Gb3 Analyses

The Lyso-Gb3 level in plasma, heart, liver, and kidney samples was measured using a liquid chromatography–tandem mass spectrometry (LC-MS/MS) approach. Briefly, homogenized samples were mixed with methanol and extracted with chloroform/methanol (2:1). The mixed samples were centrifuged at 12,000× *g* for 15 min, and 20 μL of extracted supernatant was injected into the LC-MS/MS system and separated using an acetonitrile-formic acid-methanol gradient on an XSelect Peptide HSS T3 C18 column. Using analyte peak integration, Lyso-Gb3 levels were quantified with reference to prepared standard curves. The MS conditions were set as follows: ESI mode was positive; the spray voltage was 5.5 kV; the sheath was of 40 AU; the auxiliary gas was 12 AU; the capillary temperature was 320 °C; and the collision energy was 33 eV. The following parallel reaction monitoring (PRM) transitions were monitored, with a dwell time of 40 ms: 786.4 > 282.2 (Lyso-Gb3). The mobile phase A consisted of 5% ACN, 95% water, and 0.2% FA, and the mobile phase B consisted of ACN and 0.2% FA. The flow rate was set at 0.3 mL/min. The two mobile phases formed the following gradient: 0~2 min (50%, B), 2~8 min (90%, B), and 8.1~10 min (5%, B).

### 2.9. Metabonomic Analyses

To precipitate plasma proteins, 50 μL of plasma was mixed with 200 μL of methanol, vortexed for 20 s, and left on ice for 30 min. The mixed samples were centrifuged at 15,000× *g* for 20 min, and the supernatant was then analyzed using an HPLC-QE Orbitrap-MS/MS approach.

The HPLC system used for this study consisted of an HSS T3 column coupled to an Orbitrap MS instrument (Thermo Fisher Scientific, Waltham, MA, USA). The mobile phase for this study consisted of 0.1% formic acid in water (A) and acetonitrile (B), and the gradient elution settings were as follows: 0 min, 2% B; 1 min, 2% B; 18 min, 100% B; 22 min, 100% B; 22.1 min, 2% B; 25 min, 2% B. A flow rate of 0.3 mL/min was used with an injection volume of 5 μL. During LC/MS experiments, the QE mass spectrometer was used to acquire MS/MS spectra on an information-dependent acquisition (IDA) basis. Electrospray ionization source settings were as follows: Aux gas flow rate = 16 Arb; full ms resolution = 70,000; collision energy = 25 eV in the NCE model; MS/MS resolution = 17,500; and spray voltage = −3.0 kV or 3.6 kV.

Metabolite quantification was performed by using the ABF Converter software (http://www.reifycs.com/AbfConverter/, accessed on 1 December 2023) to convert raw data into “Analysis Base File” (ABF) files. For data processing, the MSDIAL 2.2.62 software was used for peak detection, deconvolution, and alignment. SIMCA (version 14.1) was used to import the obtained data. Principal component analysis (PCA) and orthogonal partial least squares discrimination analysis (OPLS-DA) analyses were performed on the SIMCA data. Mass values of differential metabolites were retrieved from the Human Metabolome Database (HMDB). MetabolAnalyst 5.0 was used to analyze differential metabolites in clusters and pathway analyses.

### 2.10. Statistical Analysis

Statistical analyses were performed using SAS 9.2 for Windows (SAS Institute Inc., Cary, NC, USA). All data were presented as means with standard deviations and analyzed using one-way ANOVAs and Duncan’s multiple range test when more than two groups were compared, while the student’s *t*-test was utilized when only two groups were compared. Statistical significance was defined as *p* < 0.05.

## 3. Results

### 3.1. α-Gal A mRNA-LNP Administration Alters Enzyme Expression and Activity

**Figure 1:**
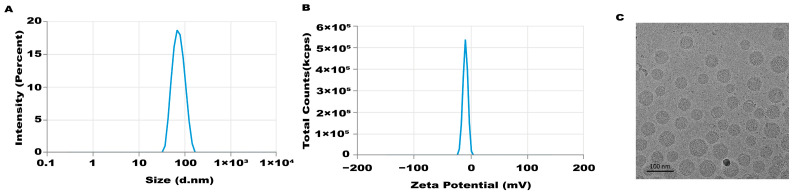
Characteristics of LNP-mediated α-Gal A mRNA formulation. Particle size (**A**), potential (**B**), and morphology (**C**) of the LNP-mediated α-Gal A mRNA formulation were analyzed.

**Figure 2:**
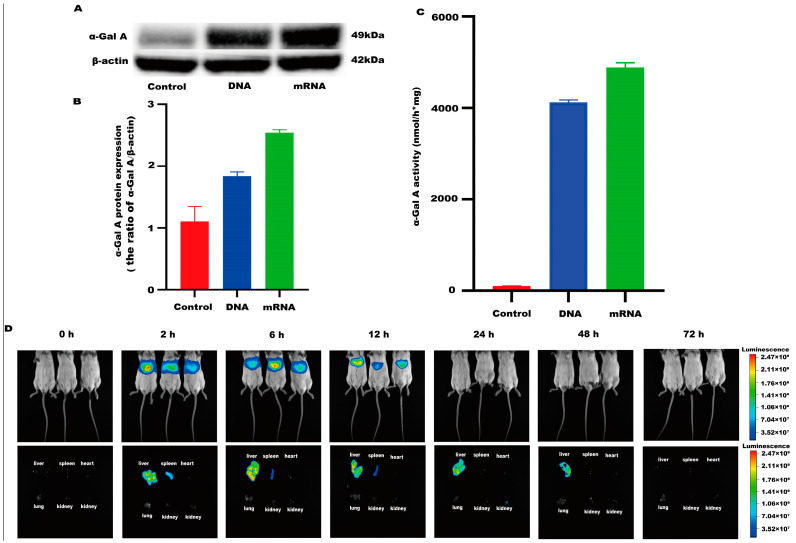
α-Gal A activity and expression level in HEK293 cells. (**A**) Western blotting was used to detect the expression of α-Gal A in HEK293 cells. (**B**) The α-Gal A protein expression was calculated in HEK293 cells (*n* = 3). (**C**) ELISA was used to measure α-Gal A enzyme activity in HEK293 cell lysates (*n* = 6). DNA plasmid was transfected into HEK293 cells as a positive control. (**D**) Expression time and tissue distribution of LNP-encapsulated luciferase (1 mg/kg) in wild-type mice (*n* = 3).

An mRNA-LNP preparation was produced by encapsulating this in vitro transcribed mRNA in LNPs. The characterization of this preparation revealed an mRNA concentration of 877.02 μg/mL and an average particle size of 70.30 nm. The potential of this preparation was −4.893 mV (Figure 1A–C), and the encapsulation rate was 93.4%. The in vitro transcribed mRNA product efficiently promoted α-Gal A expression in HEK293 cells, both in the mRNA (Figure 2A) and protein levels (Figure 2B). An α-Gal ELISA kit was then used to analyze the activity of α-Gal A. As shown in Figure 2C, the α-Gal enzyme activity in the mRNA group was significantly increased compared to the control group. A similar result was observed in the cell lysate (Supplement Figure S1A).

**Figure 3:**
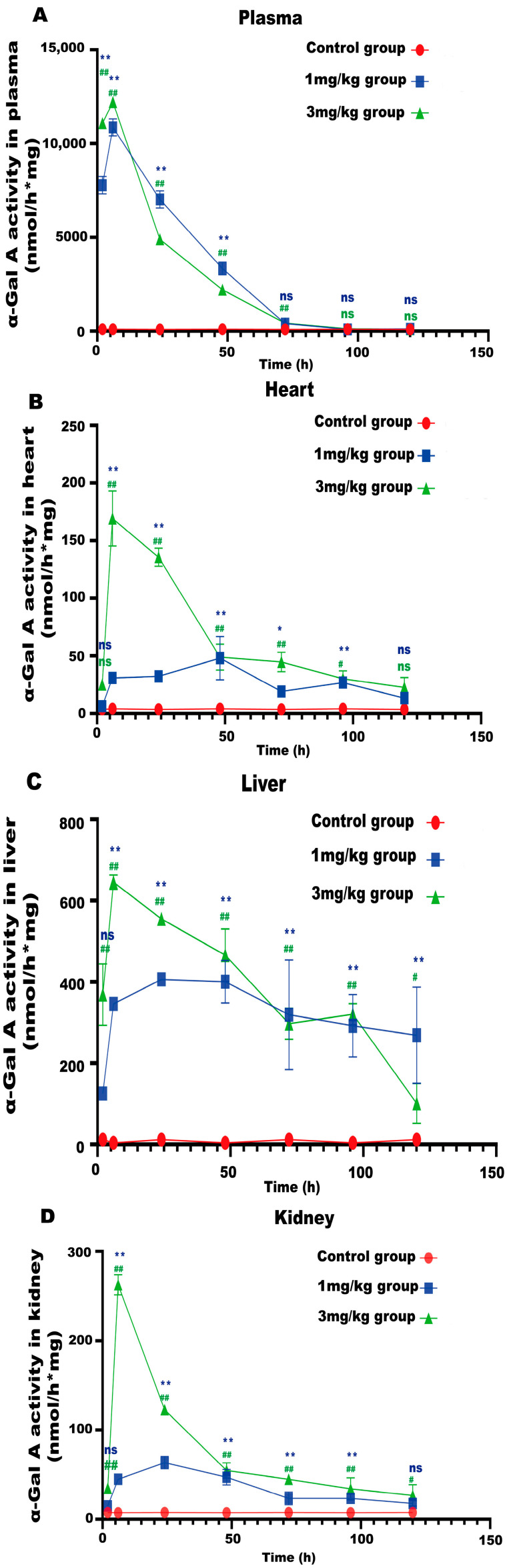

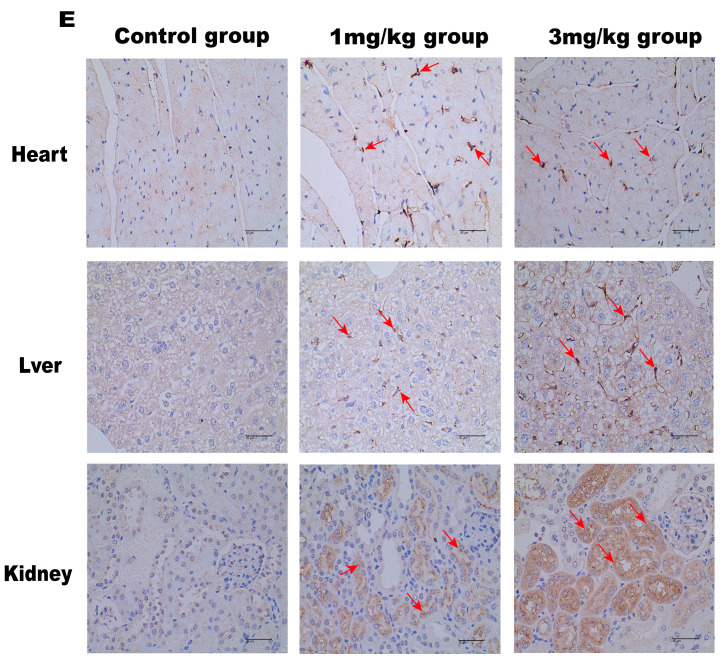
α-Gal A activity in Fabry disease model mice. ELISAs were used to detect α-Gal A activity in the plasma (**A**), heart (**B**), liver (**C**), and kidneys (**D**) of Fabry disease model mice (*n* = 6). (**E**) α-Gal A protein expression in various organs and tissues of Fabry disease model mice. Scale bar =  40 μm. The red arrows indicate expression of the α-Gal A protein. Values are presented as the means ± standard deviation (SD). ns represents not significant. The * indicates that the 1 mg/kg group has statistical significance compared to the control group (* *p* < 0.05, ** *p* < 0.01), while the # indicates that the 3 mg/kg group has statistical significance compared to the control group (# *p* < 0.05, ## *p* < 0.01).

LNP encoding luciferase (1 mg/kg) was administered via tail vein injection to wild-type mice, and it was shown that LNP-encapsulated luciferase was continuously expressed in mice for 48 h, mainly in the liver, with a small portion expressed in the spleen, kidneys, and heart (Figure 2D). When *GLA* mRNA was injected into the tail vein of wild-type mice, no difference in enzyme activity was observed between the injected group and the control group (Supplement Figure S1B). On the other hand, injection of the mRNA into the Fabry disease model mice dramatically increased the α-Gal A activity. Plasma α-Gal A activity in both the low-dose group (1 mg/kg) and the high-dose group (3 mg/kg) increased significantly within 6 h post-injection and remained at high levels from 6–24 h (Figure 3A). The activity of α-Gal A in each group began to decline after 24 h. Similarly, the α-Gal A activity in the kidney, liver, and heart of the mice in each group was significantly increased compared to the control group (Figure 3B–D). Immunohistochemical analyses also confirmed that α-Gal A protein levels in the heart, liver, and kidneys were enhanced at 24 h post-administration (Figure 3E).

### 3.2. α-Gal A mRNA Treatment Reduces Lyso-Gb3 Level and Attenuates the Hepatic Inflammatory Response in Fabry Model Mice

**Figure 4:**
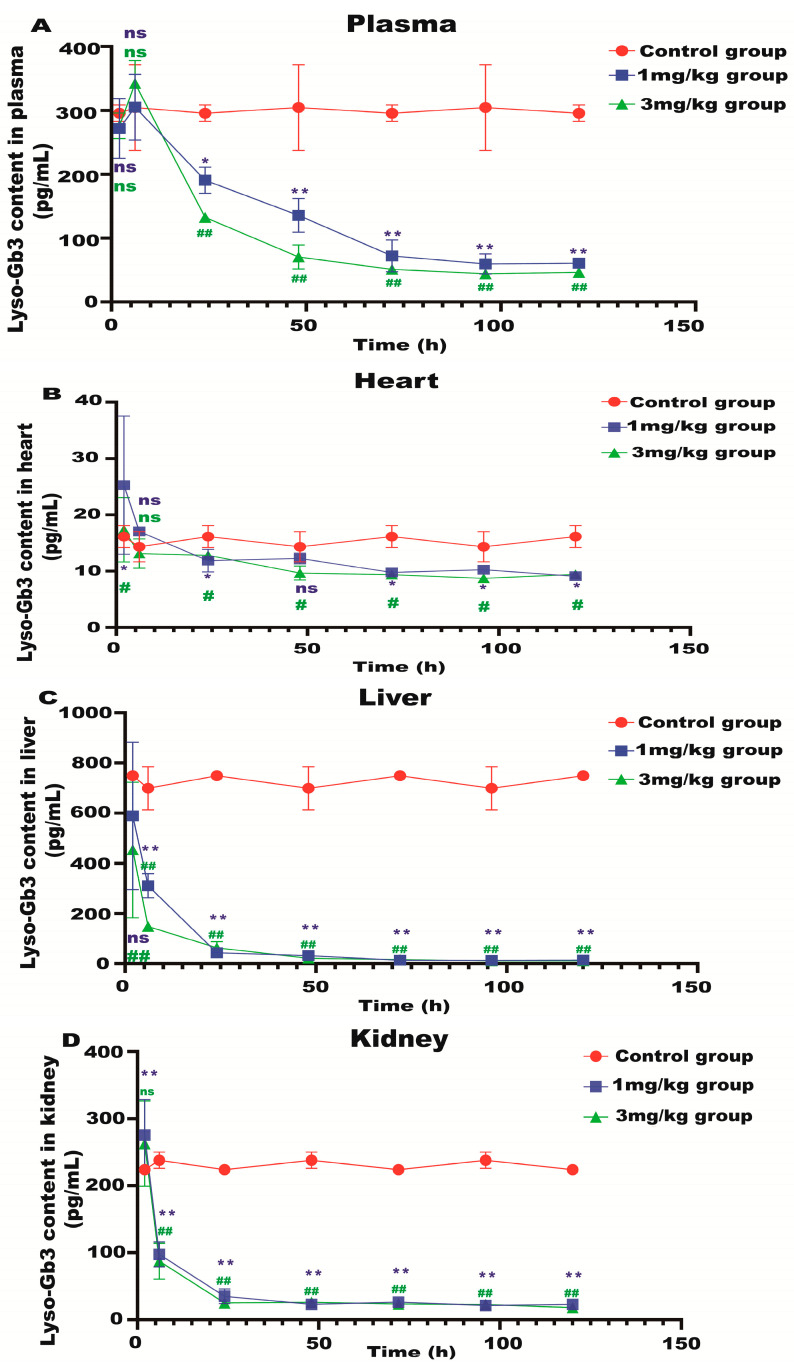

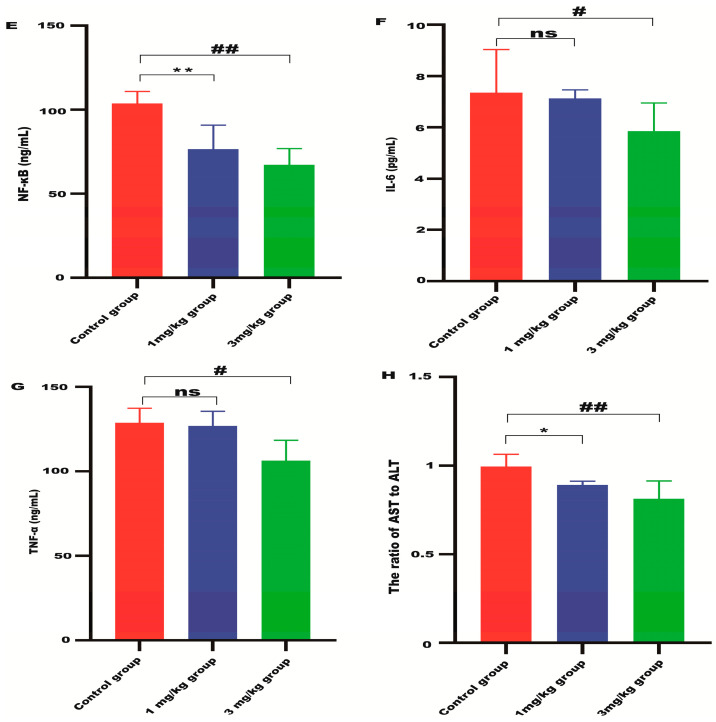
Lyso-Gb3 level in Fabry disease model mice. HPLC-MS/MS was used to detect the level of Lyso-Gb3 in plasma (**A**), heart (**B**), liver (**C**), and kidneys (**D**) of Fabry disease model mice (*n* = 6). ELISAs were used to detect the levels of NF-κB (**E**), IL-6 (**F**), TNF-α (**G**), and AST to ALT (**H**) in Fabry disease model mice. Values (*n* = 6) are presented as the means ± SD. ns represents not significant. The * indicates that the 1 mg/kg group has statistical significance compared to the control group (* *p* < 0.05, ** *p* < 0.01), while the # indicates that the 3 mg/kg group has statistical significance compared to the control group (# *p* < 0.05, ## *p* < 0.01).

Lyso-Gb3 is a biomarker for Fabry disease. When Lyso-Gb3 levels in the plasma, heart, liver, and kidneys of the Fabry disease model mice in each treatment group were analyzed using HPLC-MS/MS, it was found that the Lyso-Gb3 level of the mRNA administration groups was significantly reduced compared to the control group (Figure 4A–D), confirming the in vivo efficacy of α-Gal A mRNA treatment.

ALT and AST are indicators of liver damage. The ratio of AST to ALT in the liver of the mRNA-treated mice was significantly reduced compared with the control group (Figure 4H), indicating that α-Gal A mRNA delivery can alleviate liver damage in Fabry disease model mice. The level of inflammatory cytokines, including NF-κB, IL-6, and TNF-α, was also significantly suppressed in the liver of animals treated with high-dose α-Gal A mRNA (Figure 4E–G). These results indicated that α-Gal A mRNA attenuated hepatic inflammation in these model mice.

### 3.3. Metabolomic Analyses of the Plasma of Fabry Disease Model Mice

After validating the efficacy of the α-Gal A mRNA, an HPLC-MS/MS approach was used to analyze the metabolic profile of murine plasma samples after the treatment. An unsupervised PCA approach was used to analyze the experimental data of the treated and untreated control groups. The first two principal components explained 73.7% and 64.7% of the total variance in both positive and negative ion modes. PCA plots revealed clear separation among the three groups in both positive and negative ion modes (Supplement Figure S2A,B,G,H). The six QC samples clustered together, demonstrating good instrument stability throughout these HPLC-MS/MS analyses. The use of OPLS-DA plots further increased metabolite separation between the control and experimental groups. Based on these OPLS-DA score plots, there was a clear difference between the control group and other groups in both positive and negative ion modes (Supplement Figure S2C–F,I–L). As shown by the R2X, R2Y, and Q2 values, this OPLS-DA model was robust and predictive.

**Figure 5:**
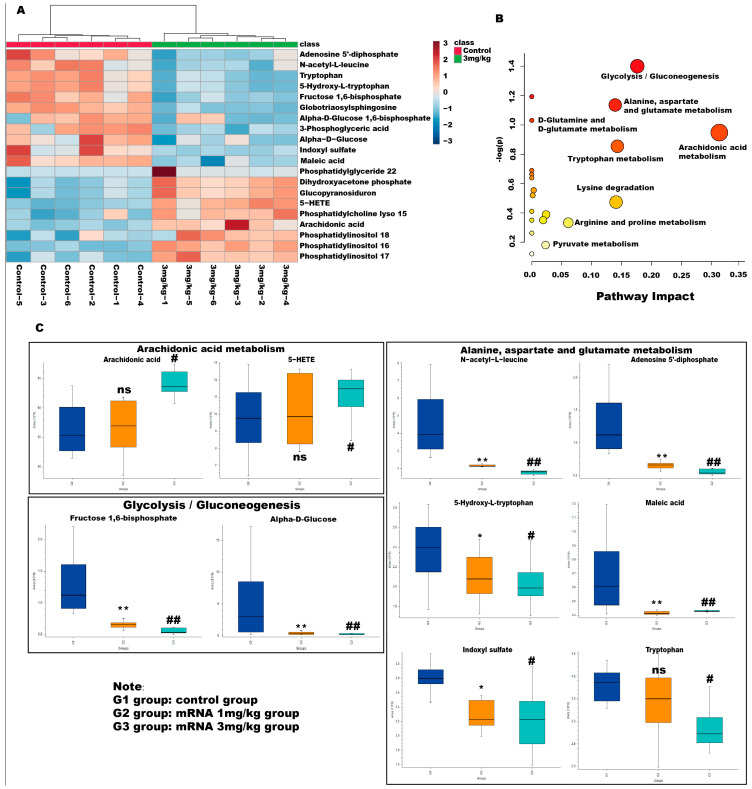
Metabolic pathway analyses of differential metabolites in Fabry disease model mice. (**A**) Enrichment analysis heat map of differential metabolites in different groups. Heatmap represents the median-normalized log2 intensity for each analyte, scaled across all samples. Red indicates relative enrichment, whereas blue indicates relative depletion. (**B**) Enrichment maps of differential metabolites in different metabolic pathways. The horizontal axis represents the degree of influence of the enriched pathway of differential metabolites. The vertical axis represents the *p*-value of the enriched pathway. *p*-value was determined by Student’s *t*-test. The *p*-value represents significance, and the larger the –log (*p*), the greater the significance of the metabolic pathway. (**C**) Relative level of changes for different differential metabolites between groups in the arachidonic acid metabolism, alanine, aspartate and glutamate metabolism, and glycolysis/gluconeogenesis pathway. ns represents not significant. The * indicates that the G2 group has statistical significance compared to the G1 group (* *p* < 0.05, ** *p* < 0.01), while the # indicates that the G3 group has statistical significance compared to the G1 group (# *p* < 0.05, ## *p* < 0.01).

Variable importance in the projection (VIP) values and *p*-values were used to identify differentially abundant metabolites contributing to the observed separation. Potential differential metabolites were screened based on the following criteria: VIP > l and *p* > 0.05. As shown in Table 1, 20 potential metabolites were identified. We further conducted thermographic enrichment analysis on these differential metabolites (Figure 5A). The heat map revealed that the administration of mRNA significantly increased the level of metabolites such as dihydroxyacetone phosphate, glucopyranosiduron, 5-HETE, phosphatidylcholine lyso 15, arachidonic acid, phosphatidylinositol 18, phosphatidylinositol 16, and phosphatidylinositol 17, which correspond to inositol phosphate metabolism, phosphatidylinositol signaling system, and arachidonic acid metabolism. On the other hand, metabolites such as adenosine 5’-diphosphate, N-acetyl-L-leucine, tryptophan, 5-hydroxy-L-tryptophan, fructose 1,6-bisphosphate, globotriaosylsphingosine, alpha-D-bisphosphate, 3-phospho-D-glycerate, alpha-D-Glucose, indoxyl sulfate, maleic acid, which correspond to fructose and mannose metabolism, glycolysis/gluconeogenesis and tryptophan metabolism, exhibited a significantly reduced level in the treated group (Figure 5C). These metabolites are related to amino acid metabolism and energy metabolism pathways, which are commonly considered to be altered in Fabry diseases.

Metabolic pathways associated with these differential metabolites were then analyzed with MetaboAnalyst 5.0. As shown in Figure 5B, the pathway that exhibited the highest impact after mRNA administration is the arachidonic acid metabolism and glycolysis/gluconeogenesis, followed by alanine, aspartate and glutamate metabolism, and tryptophan metabolism pathways, suggesting that mRNA can effectively affect the metabolism of amino acids and energy in Fabry disease and can quickly degrade glycosaminolipids represented by Lyso-Gb3 in the body. Interestingly, the levels of arachidonic acid and 5-HETE showed a significant change in the high-dose administered group but not in the low-dose group, which is likely due to the impact of mRNA-encapsulated LNP in the high-dose group mice.

## 4. Discussion

Fabry disease is an X-linked lysosomal storage disorder caused by mutations in the *GLA* gene encoding α-Gal A. Glycosphingolipids, particularly Gb3 and Lyso-Gb3, accumulate in the context of α-Gal A deficiency [2]. After the administration of α-Gal mRNA, the activity of α-Gal A significantly increased, which resulted in a reduction in the Lyso-Gb3 level in the plasma, heart, liver, and kidney of the Fabry disease model mice. The levels of Lyso-Gb3 remained low in the Fabry disease mice at 120 h post-administration, highlighting a prolonged α-Gal mRNA therapeutic window. Compared to the study of Zhu et al. [10], the duration time of our present study is much shorter. The main reason is that mRNA encapsulated by LNP can be metabolized by the body in a relatively short period of time, and as our goal is to explore the effects of messenger ribonucleic acid drugs on the body’s metabolic pathways, we decided to evaluate the metabolome of treated mice within a shorter time frame after the administration of the RNA drug. However, it will be of interest to further investigate metabolomic changes over a longer period, which will shed light on the duration and long-term effects of the mRNA on the metabolome of the entire body.

Lipid accumulation in the liver induces hepatic infiltration by immune cells and consequent inflammation [14]. Given that α-Gal A activity was detected in the liver of Fabry disease model mice following the intravenous injection of α-Gal A mRNA, the activity of liver transaminases was quantified to assess hepatic injury. Compared with control mice, α-Gal A mRNA treatment resulted in a significant decrease in the AST-to-ALT ratio in the murine liver. Fabry disease is characterized by a chronic inflammatory response, of which the accumulation of Lyso-Gb3 and Gb3, lactosylceramide, glucosylceramide, and other substrates causes an inflammatory response in the liver and other organs. Our current study demonstrated that even low-dose α-Gal A mRNA administration was sufficient to significantly reduce the level of NF-κB in the mouse liver, suggesting that the mRNA can efficiently abrogate liver inflammation in Fabry disease model mice.

HPLC-MS/MS-based metabolomic analysis revealed marked changes in the plasma metabolite profiles of Fabry disease mice following α-Gal A mRNA administration. In addition to the changes in Lyso-Gb3 levels, 19 metabolites exhibited significant changes in abundance following the application of α-Gal A mRNA. Isoprostanes are generated following the peroxidation of arachidonic acid and arachidonic esters of phospholipids in the context of inflammation. In the treatment groups, significant changes in arachidonic acid and phosphatidylinositol levels were noted, demonstrating that such treatment was sufficient to regulate inflammation governed by the phosphatidylinositol signaling system and arachidonic acid metabolism. Interestingly, there is also literature indicating that elevated levels of arachidonic acid and 5-HETE promote inflammatory responses. The increase in arachidonic acid level can promote the elevation of prostaglandin G2, which further raises the level of prostaglandin E2, thereby strengthening the occurrence and development of inflammation [15]. The reason for the above situation needs to be investigated further. However, a possible reason may be due to the presence of a large amount of lipids in the LNP components.

Metabolites that are significantly enriched in the glycolysis/gluconeogenesis pathways, including alpha-D-glucose, alpha-D-Glucose 1,6-bisphosphate, and fructose 1,6-bisphosphat,e were found to decrease greatly in α-Gal A mRNA-treated mice. Although these energy metabolism-related compounds are generally considered to be suppressed in Fabry model cells [16], it was also demonstrated that the energy metabolism was shifted toward glycolysis from fatty acid β-oxidation in Fabry model cells, suggesting that the deficiency of the *GLA* gene can result in enhanced glycolysis. Similarly, metabolites that are related to amino acid metabolism were reduced in the treatment group. Amino acid metabolism is interwound with carbohydrate and fatty acid metabolisms. Hence, the interference of one metabolic pathway will likely directly or indirectly affect others. We speculate that the recovery of lipid metabolism in the treatment group led to the balancing of the other metabolic pathways, which exhibited a suppressive effect. Such an unexpected profile highlights the necessity of evaluating the consequences of mRNA administration in the in vivo system, as the metabolic processes in the body may differ from those in the cellular model, and different metabolic pathways need to cross-talk to each other and maintain the homeostasis of the full system.

The malfunction of lipid metabolism is a major phenomenon in Fabry disease. However, only two related metabolites, i.e., globotriaosylsphingosine and phosphatidylglyceride 22, were found to be altered in our present metabolomic analysis. Phosphatidylglyceride 22 is not only a component of biomembranes but is also involved in the formation of bile and membrane surfactants and participates in the recognition and signal transduction processes. Its level was found to rapidly accumulate in the bodies of Fabry patients [17]. The level of phospholipid was also increased in our treatment group, which is different from previous research results. It is likely caused by the presence of cholesterol in the LNP that encapsulates mRNA. Additionally, the lack of major changes in lipid metabolism observed in our current study may be due to the relatively short duration of the experiment. Fabry disease is a lifelong illness, and a significant change in the profile of lipid-related metabolites may require a longer treatment period and possibly multiple injections. A future long-term drug administration study will be needed to clarify this issue.

Compared with the control group, we found that the levels of phosphatidylinositol 16, 17, and 18 were significantly increased in the treatment group. Phosphatidylinositol is composed of 1,2-diacylglycerol phosphate and inositol. Phosphatidylinositol plays a crucial role in cell morphology, metabolic regulation, signal transduction, and various physiological functions [17]. In terms of signal transduction, phosphatidylinositol transfer protein and phosphatidylinositol are important upstream regulatory factors involved in the regulation of the Hippo signaling pathway [18] and the phosphatidylinositol 3-kinase (PI3K) signaling pathway. Studies have shown that the PI3K signaling pathway plays an important role in regulating cellular lipid metabolism [19]. The significant increase in phosphatidylinositol in the treatment group is likely due to the activation of the PI3K signaling pathway, which further regulates lipid metabolism and related pathways. Further study is warranted to clarify whether the PI3K signaling pathway is activated in mRNA-treated mice and involved in the regulation of lipid metabolism.

Studies have shown that polyunsaturated fatty acids are sensitive to oxidation [20], and low-density lipoprotein oxidation is a critical step in the development of atherosclerosis [21]. Fabry disease patients harbor increased lipid, protein oxidation, and inflammation levels, together with decreased levels of antioxidant defenses such as heme oxygenase 1 [22]. Moreover, endothelial dysfunction was reported in Fabry disease patients [23]. Oxidative stress was initially assumed in Fabry disease based on elevated markers of inflammation and oxidative damage. Shen et al. observed a positive association between Gb3 levels and increased production of reactive oxygen species in cultured vascular endothelial cells [24]. Furthermore, it was observed that endothelial cells exposed to plasma obtained from individuals with Fabry disease exhibited a significantly elevated production of reactive oxygen species in comparison to those treated with plasma derived from healthy individuals. The greatly suppressed level of polyunsaturated fatty acid in the mRNA-treated mice hence suggested that the therapeutic agent not only recovered the function of α-Gal A, but may also improve the redox environment in vivo. However, how such a change affects the oxidative status of Fabry disease mice remains unclear.

LNP is a novel drug delivery system that can safely and effectively deliver nucleic acids, such as DNA or RNA, to target cells. Compared with other nano-drug delivery systems, LNP has a higher nucleic acid encapsulation rate and transfection capability. In the meantime, LNP has lower cytotoxicity and immunogenicity compared to viral vectors. However, there have been reports indicating that cationic lipids have potential toxicity and immunogenicity [25]. Further, the storage conditions of LNP are relatively harsh, and the stability is poor. LNP is generally composed of ionizable lipids, phospholipids, cholesterol, and polyethylene glycol (PEG) lipids. Studies have shown that LNP is mainly distributed in the liver (81%), and the expression level can reach up to 96% through intravenous injection into mice [26]. We injected luciferase LNP into mice via intravenous injection to study its tissue distribution. The result showed that the luciferase LNP continued to be expressed for 48 h, and the majority of luciferases are distributed in the liver, with a small portion also found in the spleen, kidneys, and heart. In fact, it has been demonstrated that by changing the proportion of LNP, the LNP-mRNA can be accurately transported to organs such as the lungs, spleen, and liver of mice [27]. On the other hand, due to the presence of lipids in LNP, injection into the body may affect the metabolism of endogenous lipids. Hence, long-term and more systematic investigations such as monthly dosing cycles, application of different dosages, and long-term metabolomics studies of urine in addition to plasma will need to be carried out to understand and evaluate the LNP-mRNA system more thoroughly in in vivo Fabry’s disease systems.

**Figure 6:**
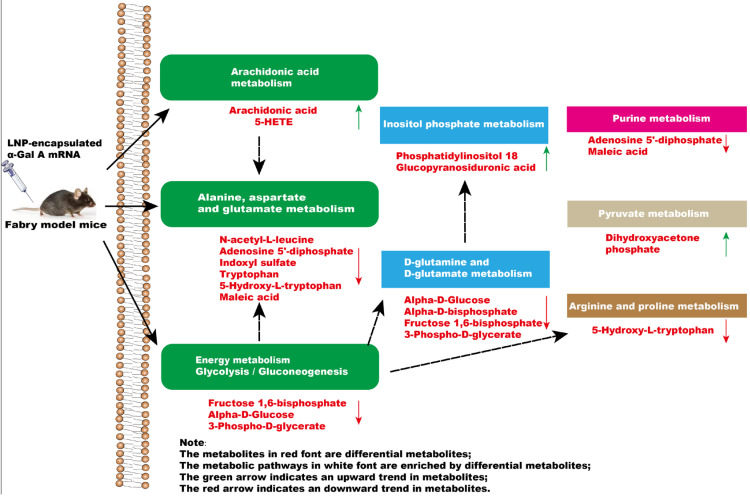
The effect of intravenous injection of α-Gal A mRNA on plasma metabolic pathways in Fabry model mice.

In summary, a significant impact occurs in the glycolysis/gluconeogenesis pathways, amino acid metabolism, and arachidonic acid metabolism of mice after administration of α-Gal A mRNA (Figure 6). Our results highlighted the efficacy of α-Gal A mRNA treatment, emphasizing its potential as a therapeutic intervention for the management of Fabry disease. Moreover, metabolomics analytical techniques were applied to investigate the metabolome alterations in Fabry disease model mice after mRNA nucleic acid injection, providing the basis for a more comprehensive evaluation of the efficacy and safety of this kind of novel treatment in the future.

## 5. Conclusions

We herein developed and validated the activity of an mRNA construct encoding α-Gal A using both a cellular system and a mice in vivo model. Metabolomic analysis of the murine plasma revealed a significant change in the glycolysis/gluconeogenesis pathways, amino acid metabolism, and arachidonic acid pathways of the mRNA-treated mice. Such information improves our understanding of the effect of mRNA therapeutic agents on the metabolism of treated subjects and comprises a preclinical proof-of-concept supporting the value of α-Gal A mRNA as a new therapeutic approach for Fabry disease patients.

## Figures and Tables

**Table 1:** Identification, fold-change values, and metabolic pathways associated with differential metabolites in the plasma of Fabry disease model mice.

NO	Formula	Metabolites	m/z	Fold Change mRNA/Control
1	C_20_H_31_O_2_•Na	Arachidonic acid	303.233	3.84
2	C_20_H_32_O_3_	5-HETE	319.228	1.37
3	C_8_H_15_NO_3_	N-acetyl-L-leucine	172.097	0.55
4	C_10_H_15_N_5_O_10_P_2_	Adenosine 5’-diphosphate	410.033	0.47
5	C_8_H_6_NO_4_SK	Indoxyl sulfate	212.002	0.46
6	C_11_H_12_N_2_O_2_	Tryptophan	203.082	0.39
7	C_11_H_12_N_2_O_2_	5-Hydroxy-L-tryptophan	219.077	0.36
8	C_4_H_4_O_4_	Maleic acid	115.002	0.33
9	C_45_H_80_O_12_P•Na	Phosphatidylinositol 16	857.52	2.38
10	C_38_H_75_O_10_PNH_3_	Phosphatidylglyceride 22	865.5	1.63
11	C_10_H_17_N_3_O_6_S	Alpha-D-Glucose	611.144	0.46
12	C_6_H_14_O_12_P_2_	alpha-D-Glucose 1,6-bisphosphate	340.115	0.29
13	C_6_H_15_NaO_12_P_2_	Fructose 1,6-bisphosphate	338.988	0.43
14	C_3_H_8_O_3_	3-Phosphoglyceric acid	184.985	0.46
15	C_46_H_80_O_13_P•Na	Phosphatidylinositol 18	883.539	5.55
16	C48H78O18	Glucopyranosiduronic acid	941.516	1.44
17	C_24_H_50_NO_7_P	Phosphatidylcholine lyso 15	512.334	1.78
18	C_3_H_7_O_6_P	Dihydroxyacetone phosphate	168.988	1.44
19	C_36_H_67_NO_17_	Globotriaosylsphingosine	785.914	0.33
20	C_45_H_87_O_13_P	Phosphatidylinositol 17	871.537	2.85

Metabolites identified in both positive and negative ion modes; m/z, mass to charge ratio. mRNA/control: the mRNA high dose group compared with the control group.

## Data Availability

Data are contained within the article and Supplementary Materials.

## Supplementary Materials

The following supporting information can be downloaded at https://www.mdpi.com/article/10.3390/biology13020106/s1. Figure S1: α-Gal A enzyme activity in HEK293 cells, wild-type mice, and KO mice. Figure S2: Metabolomic analysis of the plasma of Fabry disease mice administered with α-Gal A mRNA.
