# Supplementary figures and images for: Evaluating the Metabolic Basis of α-Gal A mRNA Therapy for Fabry Disease
Source: Biology (Basel). 2024 Feb 8;13(2):106. doi: 10.3390/biology13020106 (PMC10886685; doi:10.3390/biology13020106)

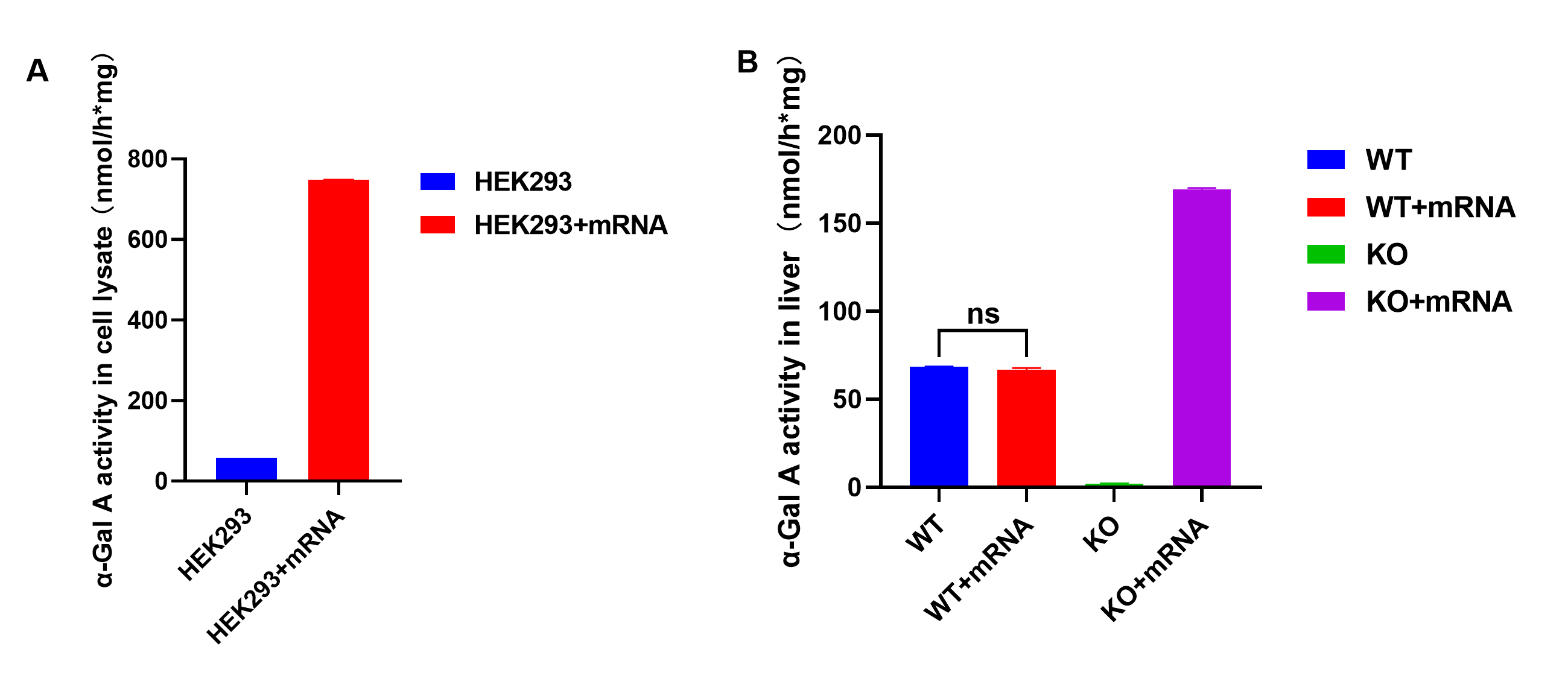

Supplement: Supplementary file 1 [file biology-13-00106-s001.zip › Figure supplement 1.tif]

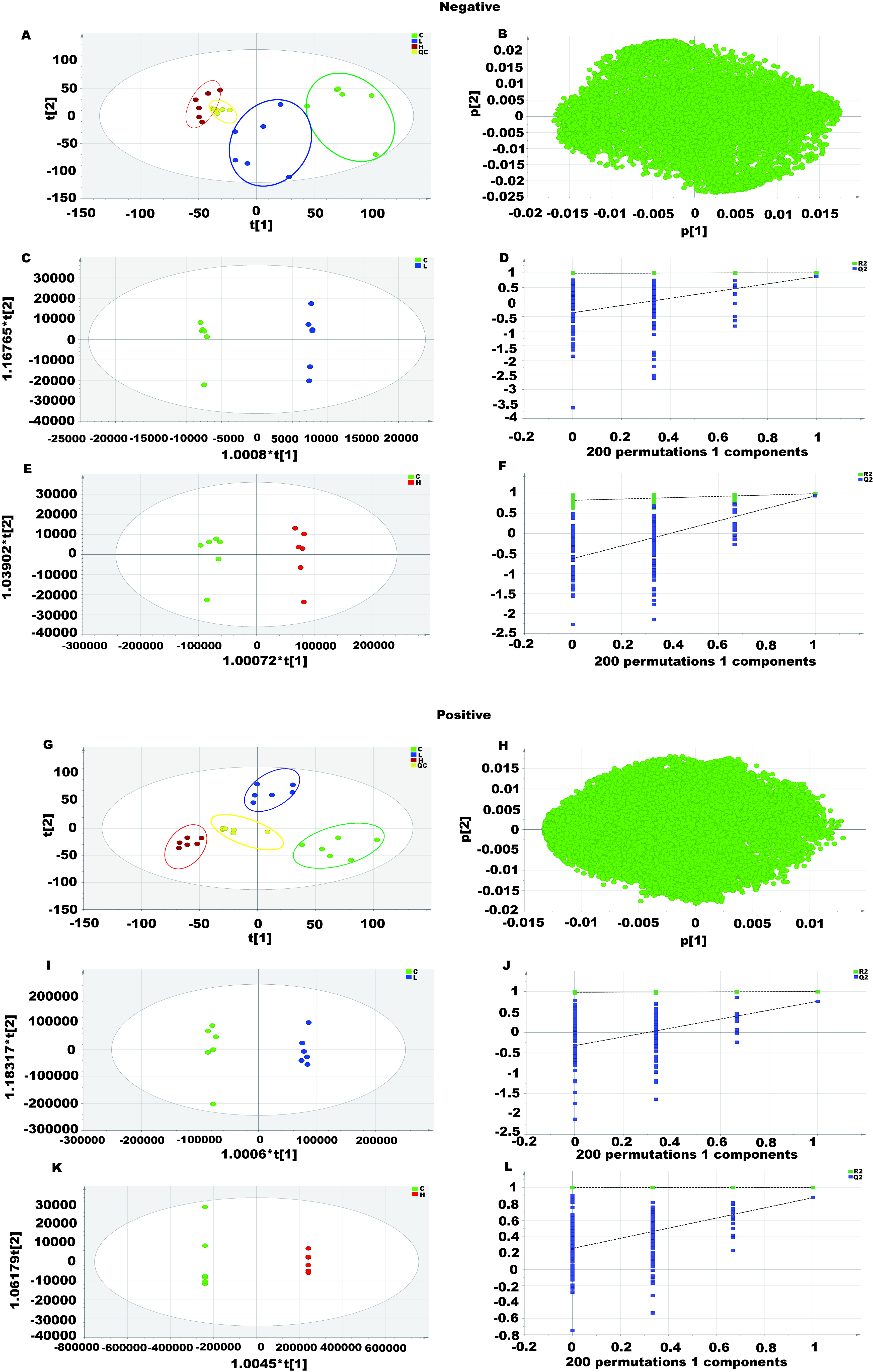

Supplement: Supplementary file 1 [file biology-13-00106-s001.zip › Figure supplement 2.tif]
